# Recurrence of Subdural Haematoma in a Population-Based Cohort – Risks and Predictive Factors

**DOI:** 10.1371/journal.pone.0140450

**Published:** 2015-10-14

**Authors:** Linnea Schmidt, Sanne Gørtz, Jan Wohlfahrt, Mads Melbye, Tina Noergaard Munch

**Affiliations:** 1 Department of Epidemiology Research, Statens Serum Institut, Copenhagen, Denmark; 2 Department of Neurosurgery, Copenhagen University Hospital, Denmark; Heinrich-Heine University, GERMANY

## Abstract

**Objectives:**

To estimate the risks of and identify predictors for recurrent subdural haematoma in surgically and conservatively treated patients.

**Methods:**

The cohort comprised all individuals diagnosed with a first-time subdural hematoma in Denmark 1996–2011. Information on potential predictors was retrieved from the Danish health registers. Cumulative recurrence risks were estimated using the Aalen-Johansen estimator. Rate ratios (RR) were estimated using Poisson regression.

**Results:**

Among 10,158 individuals with a subdural hematoma, 1,555 had a recurrent event. The cumulative risk of recurrent subdural hematoma was 9% at 4 weeks after the primary bleeding, increasing to and stabilising at 14% after one year. Predictors associated with recurrence were: Male sex (RR 1.60, 95% CI:1.43–1.80), older age (>70 years compared to 20–49 years; RR 1.41, 95% CI: 1.21–1.65), alcohol addiction (RR 1.20, 95% CI:1.04–1.37), surgical treatment (RR 1.76, 95% CI:1.58–1.96), trauma diagnoses (RR 1.14, 95% CI:1.03–1.27), and diabetes mellitus (RR 1.40, 95% CI:1.11–1.74). Out of a selected combination of risk factors, the highest cumulative 1-year recurrence risks for subdural hematoma of 25% (compared to 14% for all patients) was found in surgically treated males with diabetes mellitus.

**Conclusions:**

The recurrence risk of subdural hematoma is largely limited to the first year. Patient characteristics including co-morbidities greatly influence the recurrence risk of SDH, suggesting that individualized prognostic guidance and follow-up is needed.

## Introduction

Subdural haematoma (SDH) is one of the most common intracranial bleedings and frequently recur after discharge from the hospital. The reported recurrence risks of SDH in the literature of 9–33%[1–3] among selected populations of surgically treated cases are difficult to interpret and remain to be estimated as a rate or annual risk.

Whereas predictive factors for recurrence associated with the morphological features of the hematomas, anatomical location as well as surgical techniques are well described,[1;4–6] the knowledge of potential predictive factors for recurrence amongst the concomitant medical diseases is limited, although co-morbidities and chronic use of medicine are very frequent in patients suffering an SDH.

Few previous studies report that old age and head trauma significantly increase the risk of recurrent SDH,[1;7] whereas no association or conflicting results were observed regarding diabetes mellitus, alcohol addiction, cerebral infarction, statin therapy, renal diseases, chronic hepatic diseases, warfarin- and platelet inhibitor therapy.[1;6–11] Furthermore, most previous studies included surgically treated cases only, disregarding the fact that a large proportion of SDH patients are treated conservatively.

Based on the close to complete Danish health registers and an unselected, population-based cohort with up to 16 years of follow-up, we investigated the recurrence risks associated with a wide spectrum of potential predictors for recurrent SDH for both surgically- and conservatively treated patients.

## Materials and Methods

### Data Sources

The Danish Civil Registration System has assigned a personal identification number (CRS-number) to every individual living in Denmark since 1968[12] and is continuously updated with information on vital status and emigration. The CRS-number allows cross-linkage between all Danish registers.

The National Patient Discharge Register, established in 1977, contains information on all hospital admittances with diagnoses and operations recorded according to the International Classification of Diseases.[13] Since 1995, The Danish National Prescription Register has recorded individual-level information on all prescribed medicine dispensed at community pharmacies in Denmark. The register contains drug information such as product name, anatomical therapeutic chemical classification (ATC) code, date of dispensing, number of doses and strength.[14]

Denmark has a readily accessible, free healthcare system and reporting to the national registers is mandatory and automatized.

### Study Cohort and Follow-Up

The study cohort consisted of all individuals aged 20 years or older and diagnosed during the period 1996–2011 with a first-time registered event of SDH. The affected individuals were chosen from an unselected population encompassing all individuals in Denmark. Including only diagnoses from 1996 and forward assured information on minimum one year’s use of medicine prior to the first bleeding event for all patients. SDH diagnoses for inpatients were identified in the Danish National Hospital Discharge Register (ICD-codes are shown in S1 Table). Patients with registered diagnoses of both subdural and spontaneous intracerebral bleedings at the first hospital admittance were excluded because of suspected misclassification since the occurrence of a spontaneous intracerebral haemorrhage and SDH at the same time seems unlikely. Furthermore, patients diagnosed with an intracerebral tumour or vascular malformation before or during the same admittance as for the primary SDH were excluded.

To account for re-admittances for the same haemorrhage shortly after discharge or transfer between hospital departments, a first-time event was defined as the first hospital admittance and all subsequent admittances with less than seven intermittent days. Thus, entry to the study was seven days after the last of these discharge dates. Cohort members were followed until the first of the following events: Recurrent SDH, end of the study (December 31, 2011), death or emigration. If the second haemorrhage was of the other type than the original, the individual was censored (did not contribute to follow-up) from the date of the second haemorrhage.

### Potential Predictive Factors for SDH

Potential predictive factors for a recurrent SDH included gender, age, hypertension, alcohol abuse, diabetes, liver cirrhosis, renal insufficiency, use of statins, surgical treatment of the primary SDH, all diagnosed or registered no later than during the admittance for the primary SDH. Furthermore, a history of other trauma within 2 years prior to the primary SDH and Pre-packaged Daily Medication Doses (PDMD, see explanation below) by the patient’s pharmacy, both considered proxies for tendency to fall, were analysed as potential predictors.

Information on surgical treatment, renal insufficiency, chronic liver disease, alcohol addiction diagnoses, and trauma diagnoses was retrieved from the National Patient Discharge Register (ICD codes listed in S1 Table). Information on prescriptions of anti-diabetic medicine, anti-hypertensive medication, statins, and oral antabuse (Disulfiram) as well as information on PDMD (Pre-packaged Daily Medication Doses) dispensed by the patient’s pharmacy was obtained from the National Prescription Register (ACT codes in S1 Table). PDMD implies that the pharmacist divides a patient’s medicine into doses according to date and time of intake. This procedure is normally used for physically or cognitively impaired patients, indicating that these patients may be at higher risk of falls, thus leading to a higher risk of SDH. Individuals with these diagnoses and/or the underlying diseases treated with these particular types of medicine were considered continuously exposed after diagnosis or first prescriptions, as these diseases are considered chronic.

### Warfarin, Platelet Inhibitor, and NSAID Therapy

Use of warfarin and platelet inhibitor therapy at the time of the primary SDH is not regarded as a predictive factor for recurrence because of the short-term effect of these types of medicine. However, use of warfarin- and platelet inhibitor therapy timely related to the recurrent SDH may be a potential mediator. The user definitions of Warfarin-, platelet inhibitor-, and NSAID therapy can be found in the S1 Text.

### Statistical Analysis

We estimated overall cumulative risks of recurrent SDH since time of entry into the study using the Aalen-Johansen estimator, thus taking into account the competing risk of death. A log-linear Poisson regression model was used to estimate the overall rate ratios (RR’s) by each potential predictive factor. Hence, the RR for recurrent SDH reflects the recurrence rate among cohort members exposed to a potential predictor compared with the recurrence rate among non-exposed cohort members.

The RR’s for all potential predictors were adjusted for each other as well as for calendar period (1996–2000, 2001–2005, 2006–2011), time since admission for the primary SDH (0–<1 month, 1–<2 months, 2–<3 months, 3–<6 months, 6 months–<1 year, ≥1 year), and length of hospital stay for the primary bleeding (quartiles in days, 1–4, 5–8, 9–19, 20+).

In separate analyses, the main results were further adjusted for current use of warfarin- and platelet inhibitor therapy. Furthermore, after adjusting the primary results for the use of warfarin- and platelet inhibitor therapy, the RR’s were for the same reasons adjusted for the use of NSAID’s in a sub analysis. We estimated the RR’s for potential predictive factors within strata of operation and follow-up time by adding an interaction term into the model. All confidence intervals for the rate ratios are 95% likelihood ratio confidence intervals, and all tests are likelihood ratio tests with a significance level of 0.05.

### Ethics

The study was approved by The Danish Data Protection Agency. According to the Danish law, ethical approval is not required for register-based studies in Denmark.

## Results

**Fig 1:**
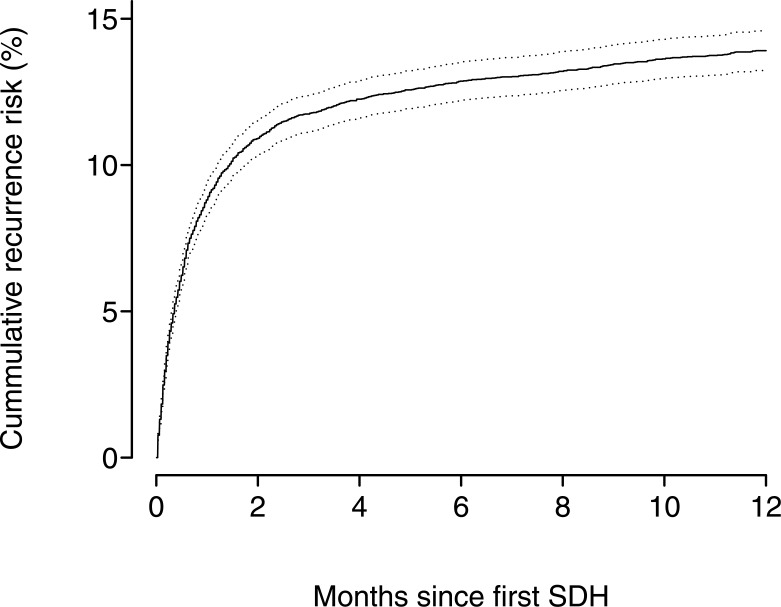
The cumulative risks in percent of recurrent subdual haematoma and intracerebral haemorrhage up to 1 year after the primary bleeding event.

**Fig 2:**
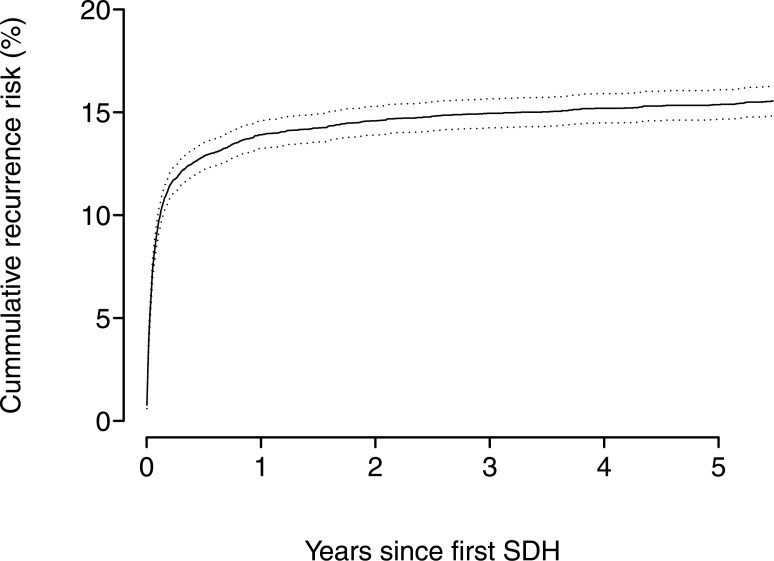
The cumulative risks in percent of recurrent subdual haematoma and intracerebral haemorrhage up to 5 years after the primary bleeding event.

Out of 10,158 cohort members diagnosed with a first-time event of SDH, 1,555 individuals experienced a recurrent SDH during follow-up. The cumulative risks in percent of recurrent SDH are presented in Figs 1 and 2, showing the cumulative risks up to 1 and 5 years after the primary bleeding event. During the first 4 weeks after the primary SDH, the cumulative risk of recurrent SDH steeply increases to a level of 9%. Thereafter the cumulative risk increased to 14% at 1 year and remained at the same level after 5-years (15%).

### Subdural Haematoma—Recurrence Risk and Predictors

The adjusted rate ratios (RR’s) and 1-year cumulative risks of the potential predictors for recurrent SDH are presented in Table 1. Males had a significantly higher recurrence risk of SDH (RR 1.60, 95% CI: 1.43–1.80) compared with females with a 1-year cumulative recurrence risk of 16.1% (females 10.0%). The RR for surgically treated compared with conservatively treated patients was 1.76, 95% CI: 1.58–1.96 with 1-year cumulative recurrence risks of 17.7% and 10.4% for surgically and conservatively treated patients, respectively. For patients with diabetes mellitus the RR was 1.35, 95% CI: 1.08–1.68 compared with other patients, with 1-year cumulative recurrence risks of 18.8% and 13.7%, respectively. Significantly higher RR’s were also observed for patients with alcohol addiction (RR 1.20, 95% CI: 1.04–1.37), and for patients with other trauma (RR 1.13, 95% CI: 1.02–1.26) compared to others, whereas these patients did not have increased 1-year cumulative recurrence risks (Table 1). The latter indicates that these factors mainly affected the long-term recurrence risks (see next section). Furthermore, age was a significant predictor for recurrent SDH with the highest observed risk among cohort members at 70 years of age or more (RR 1.41, 95% CI: 1.21–1.65).

**Table 1:** Cumulative risks and rate ratios of recurrent subdural haematoma (SDH) according to concomitant medical diseases and other potential predictors in Danish SDH patients diagnosed 1996–2011.

Potential predictors for recurrent SDH	No. of primary SDH	No. of recurrent SDH	Median follow-up time (days)	Cumulative 1-year recurrence risk in percent (95% CI) *		Adjusted RR ** (95% CI)		RR further adjusted for anticoagulation treatment*** (95% CI)	
**Age (years)**									
20–49	1895	255	1809	11.3	(9.9–12.7)	1	(ref.)	1	(ref.)
50–69	3383	511	1185	13.5	(12.4–14.7)	1.10	(0.94–1.29)	1.12	(0.96–1.31)
70-	4880	789	662	15.2	(14.2–16.2)	1.41	(1.21–1.65)	1.42	(1.22–1.67)
							P < 0.0001		
**Sex**									
Males	6575	1157	1022	16.1	(15.2–16.9)	1.60	(1.43–1.80)	1.59	(1.42–1.79)
Females	3583	398	933	10.0	(9.0–11.0)	1	(ref.)	1	(ref.)
**Alcohol addiction**									
Yes	2017	336	952	13.9	(12.4–15.4)	1.20	(1.04–1.37)	1.17	(1.02–1.34)
No	8141	1219	970	13.9	(13.2–14.7)	1	(ref.)	1	(ref.)
**Chronic liver disease**									
Yes	396	75	536	16.2	(12.5–19.8)	1.27	(0.98–1.62)	1.25	(0.97–1.59)
No	9762	1480	993	13.8	(13.1–14.5)	1	(ref.)	1	(ref.)
**Coagulopathy**									
Yes	88	15	400	16.0	(8.3–23.7)	1.19	(0.68–1.91)	1.12	(0.64–1.81)
No	10070	1540	974	13.9	(13.2–14.6)	1	(ref.)	1	(ref.)
**Surgical treatment**									
Yes	4894	957	946	17.7	(16.6–18.8)	1.76	(1.58–1.96)	1.81	(1.62–2.02)
No	5264	598	984	10.4	(9.6–11.2)	1	(ref.)	1	(ref.)
**Antihypertensive treatment**									
Yes	3286	480	660	13.6	(12.4–14.8)	0.94	(0.83–1.06)	0.97	0.86–1.10
No	6872	1075	1207	14.0	(13.2–14.9)	1	(ref.)	1	(ref.)
**Renal insufficiency**									
Yes	207	36	354	16.5	(11.4–21.6)	1.19	(0.83–1.63)	1.12	(0.79–1.54)
No	9951	1519	986	13.9	(13.2–14.5)	1	(ref.)	1	(ref.)
**Diabetes mellitus**									
Yes	487	93	414	18.8	(15.3–22.4)	1.40	(1.11–1.74)	1.35	(1.08–1.68)
No	9671	1462	1013	13.7	(13.0–14.4)	1	(ref.)	1	(ref.)
**Previous cerebral ischemic infarction**									
Yes	773	113	572	13.6	(11.2–16.1)	1.01	(0.83–1.22)	0.99	(0.81–1.20)
No	9385	1442	1013	14.0	(13.2–14.9)	1	(ref.)	1	(ref.)
**Statins**									
Yes	1145	172	556	15.0	(12.9–17.1)	0.98	(0.81–1.16)	1.05	(0.88–1.26)
No	9013	1383	1051	13.8	(13.1–14.5)	1	(ref.)	1	(ref.)
**Trauma diagnosis** ^**†**^									
Yes	6121	927	859	13.8	(13.0–14.7)	1.14	(1.03–1.27)	1.13	(1.02–1.26)
No	4037	628	1177	14.0	(13.0–15.1)	1	(ref.)	1	(ref.)
**PDMD** ^**Ω**^									
Yes	215	30	368	12.3	(7.9–16.7)	1.05	(0.71–1.49)	1.14	(0.77–1.63)
No	9943	1525	996	13.9	(13.3–14.6)	1	(ref.)	1	(ref.)

*95% Confidence interval

** Adjusted for age, sex, calendar period, time since admittance -, and length of hospital stay for the primary SDH, as well as all the listed potential predictors for recurrent SDH.

***Adjusted for the use of platelet inhibitors and warfarin.

^†^ Trauma diagnosis less than 2 years before the primary SDH.

^Ω^ PDMD: Pre-packaged Daily Medication Doses.

A total of 514 patients received Warfarin therapy at some point during follow-up (after the primary SDH), 2248 patients received ASA, and 428 patients received Clopidogrel. Further adjustment of the rate ratios for current use of platelet inhibitor- and warfarin therapy at the time of the second SDH did not change the results markedly (Table 1), neither did adjustment for the use of NSAID’s.

### Recurrence Risk and Predictors by Years Since the Primary SDH

The different predictors may carry varying risks of recurrence dependent on time since the primary SDH. Therefore, rate ratios (RR’s) for all potential predictors were estimated within the first year and after the first year following the primary SDH, presented in Table 2. Results revealed significantly stronger associations with recurrence more than one year after the primary SDH for the following predictors: alcohol addiction (RR<1 year 1.05, 95% CI: 0.91–1.22; RR≥1 year 2.68, 95% CI: 1.93–3.68 (p < .0001)), Pre-packaged Daily Medication Doses (RR<1 year 0.94, 95% CI: 0.62–1.37; RR≥1 year 4.06, 95% CI: 1.25–9.64 (p = 0.02)), and chronic liver disease RR<1 year 1.16, 95% CI: 0.88–1.50; RR≥1 year 2.71, 95% CI: 1.41–4.70 (p = 0.02)). However, the association with chronic liver disease after one year was reduced (chronic liver disease RR≥1 year 1.65, 95% CI: 0.84–2.96) when adjusted for the association with alcohol addiction after one year.

**Table 2:** Rate ratio (RR) of recurrent subdural haematoma (SDH) within the first year and after the first year of follow-up, according to concomitant medical diseases and other potential predictors in Denmark 1996–2011.

Potential predictors for recurrent SDH	<1 year			≥1 year			P-value^+^
	Events	Adjusted RR **	(95% CI*)	Events	Adjusted RR **	(95% CI*)	
**Age (years)**							
20–49	211	1	(Ref.)	44	1	(Ref.)	0.09 ^Ω^
50–69	452	1.13	(0.96–1.34)	59	0.95	(0.64–1.41)	
70-	731	1.47	(1.24–1.74)	58	1.06	(0.71–1.58)	
**Gender**							
Males	1042	1.63	(1.44–1.84)	115	1.43	(1.03–2.04)	0.50
Females	352	1	(Ref.)	46	1	(Ref.)	
**Alcohol addiction**							
Yes	275	1.05	(0.91–1.22)	61	2.68	(1.93–3.68)	< .0001
No	1119	1	(Ref.)	100	1	(Ref.)	
**Chronic liver disease**							
Yes	63	1.16	(0.88–1.50)	12	2.71	(1.41–4.70)	0.02
No	1331	1	(Ref.)	149	1	(Ref.)	
**Coagulopathy**							
Yes	14	1.17	(0.66–1.91)	1	1.31	(0.07–5.83)	0.92
No	1380	1	(Ref.)	160	1	(Ref.)	
**Surgical treatment**							
Yes	856	1.77	(1.58–1.98)	101	1.61	(1.17–2.24)	0.59
No	538	1	(Ref.)	60	1	(Ref.)	
**Antihypertensive treatment**							
Yes	440	0.92	(0.81–1.04)	40	1.13	(0.78–1.60)	0.29
No	954	1	(Ref.)	121	1	(Ref.)	
**Renal insufficiency**							
Yes	34	1.18	(0.82–1.64)	2	1.23	(0.20–3.87)	0.95
No	1360	1	(Ref.)	159	1	(Ref.)	
**Diabetes mellitus**							
Yes	90	1.44	(1.14–1.79)	3	0.76	(0.19–2.00)	0.23
No	1304	1	(Ref.)	158	1	(Ref.)	
**Previous cerebral ischemic infarction**							
Yes	103	0.99	(0.80–1.21)	10	1.15	(0.57–2.07)	0.67
No	1291	1	(Ref.)	151	1	(Ref.)	
**Statins**							
Yes	167	1.01	(0.84–1.21)	5	0.50	(0.18–1.10)	0.09
No	1227	1	(Ref.)	156	1	(Ref.)	
**Trauma diagnosis** ^**†**^							
Yes	832	1.12	(1.00–1.25)	95	1.36	(0.99–1.87)	0.25
No	562	1	(Ref.)	66	1	(Ref.)	
**PDMD** ^**††**^							
Yes	26	0.94	(0.62–1.37)	4	4.06	(1.25–9.64)	0.02
No	1368	1	(Ref.)	157	1	(Ref.)	

* 95% Confidence interval

** Rate ratios adjusted for calendar period, time since admittance for first SDH, length of hospital stay for the primary bleeding, and potential predictors for recurrent SDH listed in Table 1.

^+^ P-value: Comparison of the RR’s within and after the first year, following the primary SDH.

^Ω^ Homogeneity test

^†^ Trauma diagnosis less than 2 years before the primary SDH

^††^ PDMD: Pre-packaged Daily Medication Doses.

The higher rate ratios after the first year among some of the predictive factors, combined with the fact that the overall rate ratio and the 1-year cumulative risks were adjusted slightly differently may explain why a significant overall rate ratio was not accompanied by an elevated 1-year cumulative risk for all predictors (Table 1).

### Recurrence Risk and Predictors by Type of Treatment Modality

Table 3 shows rate ratios for recurrent SDH according to predictors in surgically- and conservatively treated cohort members. Predictive factors were similar for both treatment modalities, except for age and coagulopathy, which were more associated with recurrence in conservatively treated cohort members.

**Table 3:** Rate ratio (RR) of recurrent subdural haematoma (SDH) in surgically- and conservatively treated patients, according to potential predictors in Denmark 1996–2011.

Potential predictors for recurrent SDH	Surgical treatment			Conservative treatment			P-value^+^
	Events	Adjusted RR **	(95% CI*)	Events	Adjusted RR **	(95% CI*)	
**Age (years)**							
20–49	123	1	(Ref.)	132	1	(Ref.)	0.005
50–69	317	0.90	(0.73–1.12)	194	1.29	(1.04–1.62)	
70-	517	1.12	(0.91–1.38)	272	1.79	(1.44–2.23)	
**Gender**							
Males	743	1.69	(1.45–1.97)	414	1.50	(1.26–1.79)	0.31
Females	214	1	(Ref.)	184	1	(Ref.)	
**Alcohol addiction**							
Yes	186	1.29	(1.08–1.53)	150	1.10	(0.90–1.33)	0.20
No	771	1	(Ref.)	448	1	(Ref.)	
**Chronic liver disease**							
Yes	43	1.35	(0.96–1.83)	32	1.19	(0.81–1.68)	0.60
No	914	1	(Ref.)	566	1	(Ref.)	
**Coagulopathy**							
Yes	6	0.70	(0.28–1.42)	9	2.24	(1.07–4.10)	0.03
No	951	1	(Ref.)	589	1	(Ref.)	
**Renal insufficiency**							
Yes	18	0.92	(0.56–1.43)	18	1.65	(0.99–2.56)	0.09
No	939	1	(Ref.)	580	1	(Ref.)	
**Diabetes mellitus**							
Yes	53	1.24	(0.92–1.64)	40	1.67	(1.18–2.29)	0.18
No	904	1	(Ref.)	558	1	(Ref.)	
**Previous cerebral ischemic infarction**							
Yes	60	0.86	(0.66–1.11)	53	1.25	(0.93–1.64)	0.07
No	897	1	(Ref.)	545	1	(Ref.)	
**Statins**							
Yes	112	1.00	(0.81–1.24)	60	0.93	(0.69–1.22)	0.64
No	845	1	(Ref.)	538	1	(Ref.)	
**Trauma diagnosis** ^**†**^							
Yes	531	1.19	(1.04–1.36)	396	1.06	(0.89–1.26)	0.30
No	426	1	(Ref.)	202	1	(Ref.)	
**PDMD** ^††^							
Yes	17	1.11	(0.66–1.74)	13	0.98	(0.53–1.63)	0.74
No	940	1	(Ref.)	585	1	(Ref.)	

* 95% Confidence interval

** Rate ratios adjusted for age, gender, calendar period, time since admittance for first SDH, length of hospital stay for the primary SDH, and the potential predictors for recurrent SDH listed in Table 1.

^+^ P-value: test for similar RR’s for surgically vs conservatively treated patients for each predictive factor.

^†^ Trauma diagnosis less than 2 years before the primary SDH

^††^ PDMD: Pre-packaged Daily Medication Doses.

### Recurrence Risk by Combinations of Predictors

The 1-year cumulative recurrence risks of SDH according to selected combinations of potential predictive factors are presented in Table 4. A risk factor profile with alcohol addiction, other type(s) of trauma, and surgical treatment for the primary SDH was associated with a 1-year cumulative recurrence risk of 21% for males and 18% for females (overall 1-year cumulative recurrence risk of SDH was 14%). Surgically treated males with diabetes mellitus had a 1-year cumulative recurrence risk of 25%, whereas the corresponding risk for females was 7%. For conservatively treated males with diabetes mellitus, the cumulative 1-year recurrence risk was 20%, whereas the corresponding risk for females was 12%.

**Table 4:** Cumulative 1-year risks of recurrent SDH, according to selected combinations of predictive factors.

		Cumulative 1-year risks (95% CI)			
		Males		Females	
Alcohol addiction + trauma diagnoses	- operation	11.1%	(8.7–13.5)	12.6%	(8.4–16.9)
	+ operation	21.4%	(17.5–25.3)	17.6%	(11.4–23.7)
Diabetes mellitus	- operation	20.1%	(13.8–26.5)	11.7%	(4.4–18.9)
	+ operation	25.3%	(18.9–31.7)	7.3%	(1.1–13.7)

## Discussion

### Recurrence Risk

Previous studies have almost exclusively reported the proportion of recurrent SDH’s (9–33%), whereas little is known regarding the rate or annual risk of recurrence.[2;3] In the present study we observed that the vast majority of recurrences occurred within the first year (14%) after which the recurrence risk was very modest. In line with Mori and co-workers most of the recurrences took place only a few weeks after the primary SDH.[9]

Findings in previous, smaller risk factor studies in more selected study populations have been equivocal indeed. I this unselected population-based study of 10,158 individuals with primary SDH of which 1,555 individuals experienced a recurrence, we found surgical treatment, diabetes mellitus, alcohol addiction, other traumas, older age, and the use of PDMD (Pre-packaged Daily Medication Doses) to be predictors for recurrence. The results of the sub analyses showed the effects on SDH recurrence of these predictors were highly dependent on the time since the primary SDH. Combinations of risk factors showed that particularly surgically treated males with diabetes mellitus had a very high risk of recurrent SDH (25%).

The 1-year cumulative recurrence risk of SDH observed in our study is based on close to complete follow-up provided by the national registers, resulting in a very high detection of recurrences. On the other hand, we cannot exclude that some of the early re-admittances are due to progression of symptoms without a true recurrent bleeding. However, we were allowed access to data from a yet unpublished Danish cohort study based on reviews of the patient files and brain CT’s of 1052 patients operated for chronic SDH in the period of 2010–2012(unpublished results, Ranberg NA, Fugleholm K). The results revealed 16.3% recurrences within a year on the same or opposite site of the primary SDH. This is in line with our finding of a cumulative 1-year risk of 17.7% (95% CI: 16.6–18.8) for surgically treated cases of both acute SDH and chronic SDH.

### Predictors for Recurrence

Interestingly, we observed that surgically treated patients not only have a significantly higher short-term recurrence risk than conservatively treated patients, but also a significantly increased recurrence risk one year or more after the primary SDH. Intuitively, one would expect surgical treatment to mainly affect the short-term recurrence risk.[15;16] We speculate that the massive inflammatory reaction seen in the dura mater and the surface of the brain even after a minimally invasive burr hole operation may enhance the long-term bleeding risk.[17;18]

The association between SDH and diabetes mellitus is intriguing since diabetes mellitus is generally considered a pro-thrombotic disease.[19] The results of previous studies are very equivocal and include both positive-, negative-, and null associations.[6;7;19] Diabetes is strongly related to obesity, so hypothetically our finding may represent a higher risk of SDH with higher BMI (Body Mass Index), possibly due to higher venous pressure. Unfortunately, data on BMI were not available in this study. Given the growing incidence of diabetes and obesity worldwide, this potential association should be further investigated.

It is a common belief among clinicians, that excessive alcohol consumption increases the risk of SDH, but previous studies, although small in size and performed partially in selected populations, have been unable to support this thought. [9;11] In the present study we found a fairly strong association with a 2.7-fold increased risk of recurrent SDH after the first year in patients with alcohol addiction. The increased risks observed for patients with other traumas and in patients using PDMD (Pre-packaged Daily Medication Doses), both proxies for tendency to fall, support a traumatic component of the aetiology of chronic SDH (which is obvious for the acute SDH).[20] The observed higher recurrence risk in patients of older age is in line with previous findings[21] and is thought to rely on the fact that chronic SDH’s are more pronounced in the elderly than acute SDH’s, and chronic SDH’s are more likely to recur. Furthermore, the cerebral atrophy seen with higher age predisposes SDH because of the stretch of the bridging veins.[22]

To our knowledge, this is the most comprehensive study investigating potential predictors for SDH in a cohort consisting of both surgically- and conservatively treated patients. The free and easily accessible health care system in Denmark enabled the establishment of a study cohort from an unselected population, which minimised selection bias. The register-based information sources ruled out recall bias and provided a wide range of close to complete information. A potential limitation of the study is that we did not have access to data regarding the size and morphological characteristics of the SDH’s, which are factors that have been suggested to be of importance for recurrence in some studies.[4;6] However, these factors are not believed to be associated with the potential predictive factors investigated in this study, and are therefore not regarded as confounders.

## Conclusions

The cumulative risk of a recurrent SDH was found to be 15% and largely limited to the first year after the primary event. Co-morbidities and other patient characteristics greatly influence the recurrence risk of SDH. The very high risks observed for individuals with selected combinations of predictors suggest more individualised prognostic information and follow-up.

## Supporting Information

S1 Table Disease diagnoses and types of medicine, according to the International Classification of Diseases (ICD) and the Anatomical Therapeutic Chemical (ATC) Classification System. (DOCX)

S1 Text User definitions of warfarin-platelet inhibitor-, and NSAID therapy. (DOCX)
