# Supplementary material for: Recurrence of Subdural Haematoma in a Population-Based Cohort – Risks and Predictive Factors
Source: PLoS One. 2015 Oct 14;10(10):e0140450. doi: 10.1371/journal.pone.0140450 (PMC4605528; doi:10.1371/journal.pone.0140450)
Supplement: S1 Table — (DOCX) [file pone.0140450.s001.docx]

S1 Table: Disease diagnoses and types of medicine, according to the International Classification of Diseases (ICD) and the Anatomical Therapeutic Chemical (ATC) Classification System.

| **Outcome** | **ICD-10 codes** | **ICD-8 codes** | **ATC codes** |
| --- | --- | --- | --- |
|  | | | |
| Traumatic subdural hemorrhage | S06.5 | 852.01, 852.11, 431.01, 431.91 |  |
| **Potential predictive factors for recurrent SDH and/or ICH** | | | |
| Head trauma | S00-S09 | 850-851 |  |
| Cerebral infarction | I63 | 432-434 |  |
| Antihypertensive treatment  (defined by antihypertensive drugs; ACE-inhibitors, angiotensin receptor blockers, calcium channel blockers, beta-blockers, and diuretics) |  |  | C03, C07, C08, C09A, C09B, C09C, C09D |
| Diabetes mellitus  (defined by anti-diabetic medicine; including both insulin and oral anti-diabetic medicine) |  |  | A10 |
| Alcohol addiction  (defined by diagnosis of alcohol abuse, including intoxication diagnosis, or use of drugs for alcohol dependence) | F10, G31.2, G62.1, G72.1, I42.6, K29.2, K70, K85.2, K86.0, L27.8A, Z72.1 | 303, 57710, 57711, 5711, 29199, 29139, 29129, 29119, 57109 | N07BB |
| Renal insufficiency | N17-N19 | 5932 |  |
| Chronic hepatic diseases | K70-K77 | 571, 573 |  |
| **Exclusion diagnoses** |  |  |  |
| Intracranial tumor | C70  C71  D32.0  D33.0-2 | 191x, 19219, 19449, 22500-09, 22620-1, 22629, 22639, 22701-2, 23819, 23839 |  |
| Arteriovenous malformation | Q28.0-2 |  |  |
| **Anticoagulation treatment and NSAID’s** | | | |
| Acetylsalicylic acid |  |  | B01AC06 |
| Clopidogrel |  |  | B01AC04 |
| Warfarin |  |  | B01AA03 |
| NSAID’s |  |  | M01A |
| **Surgical procedures** |  |  |  |
| Removal of SDH | KAAD05, KAAD10 |  |  |
