# Supplementary material for: Recurrence of Subdural Haematoma in a Population-Based Cohort – Risks and Predictive Factors
Source: PLoS One. 2015 Oct 14;10(10):e0140450. doi: 10.1371/journal.pone.0140450 (PMC4605528; doi:10.1371/journal.pone.0140450)
Supplement: S1 Text — (DOCX) [file pone.0140450.s002.docx]

**S1 Text**

**The user definitions of warfarin-platelet inhibitor-, and NSAID therapy**

Cohort members were presumably treated with anticoagulation medicine if they had filled prescriptions for platelet inhibitors (clopidogrel and acetylsalicylic acid) or vitamin-K antagonists (warfarin). Use of anticoagulation medicine was considered as a time-dependent variable indicating current use. (i.e. those cohort members who experienced a recurrent bleeding were only considered exposed if they were current users at the time of the recurrent bleeding). Current use was at any time during follow-up based on the treatment period of the last refill (restricted to a maximum of 365 days) to avoid accumulation of tablets and thus an overestimation of the exposure time.

Cohort members were no longer considered current users when no refill was made within the treatment period of the latest prescription plus a number of days corresponding to 10% of the treatment period. For acetylsalicylic acid and clopidogrel the treatment period was determined based on the prescribed number of standard dosages. The standard dosage of acetylsalicylic acid for cardiovascular indications is either 75 mg or 150 mg, and regardless of strength we assumed that one tablet corresponded to one day of treatment. For clopidogrel the standard dosage is 75 mg with one tablet corresponding to one day of treatment. For warfarin, there is no standard dosage and patients are treated according to their INR (International Normalised Ratio). The daily dosage differs between 2.5 mg and 10 mg and some patients have single days with no tablet intake. To calculate treatment period for warfarin use we therefore assumed an average daily intake of 5 mg.

The platelet inhibiting side effect of NSAID’s has a duration crudely comparable to that of platelet inhibitors. For each type of NSAID’s reported to have hemorrhagic side effects, we calculated the daily dosages from the strengths of the tablets and the standard dosages recommended for chronic users. For instance, we assumed a daily intake of 1800 mg of ibuprofen if 600 mg tablets were prescribed.
